# Pathogenesis of SARS-CoV-2 and *Mycobacterium tuberculosis* Coinfection

**DOI:** 10.3389/fimmu.2022.909011

**Published:** 2022-06-16

**Authors:** Taif Shah, Zahir Shah, Nafeesa Yasmeen, Zulqarnain Baloch, Xueshan Xia

**Affiliations:** ^1^ Faculty of Life Science and Technology, Kunming University of Science and Technology, Kunming, China; ^2^ College of Veterinary Sciences, The University of Agriculture Peshawar, Peshawar, Pakistan; ^3^ College of Veterinary Medicine, South China Agricultural University, Guangzhou, China

**Keywords:** COVID-19, tuberculosis, coinfection, SARS-CoV-2-*M. tuberculosis* pathogenesis, BCG vaccination

## Abstract

Coronavirus disease-2019 (COVID-19), caused by SARS-CoV-2, is an infectious disease that poses severe threats to global public health and significant economic losses. The COVID-19 global burden is rapidly increasing, with over 246.53 million COVID-19 cases and 49.97 million deaths reported in the WHO 2021 report. People with compromised immunity, such as tuberculosis (TB) patients, are highly exposed to severe COVID-19. Both COVID-19 and TB diseases spread primarily through respiratory droplets from an infected person to a healthy person, which may cause pneumonia and cytokine storms, leading to severe respiratory disorders. The COVID-19-TB coinfection could be fatal, exacerbating the current COVID-19 pandemic apart from cellular immune deficiency, coagulation activation, myocardial infarction, and other organ dysfunction. This study aimed to assess the pathogenesis of SARS-CoV-2-*Mycobacterium tuberculosis* coinfections. We provide a brief overview of COVID19-TB coinfection and discuss SARS-CoV-2 host cellular receptors and pathogenesis. In addition, we discuss *M. tuberculosis* host cellular receptors and pathogenesis. Moreover, we highlight the impact of SARS-CoV-2 on TB patients and the pathological pathways that connect SARS-CoV-2 and *M. tuberculosis* infection. Further, we discuss the impact of BCG vaccination on SARS-CoV-2 cases coinfected with *M. tuberculosis*, as well as the diagnostic challenges associated with the coinfection.

## Introduction

Coronaviruses (CoVs) are positive single-stranded RNA viruses that cause respiratory and gastrointestinal tract infections in mammals, such as humans, amphibians, and birds. They are important members of the *Coronaviridae* subfamily, *Orthocoronavirinae*. The International Committee on Taxonomy of Viruses has classified CoVs into four important genera: α-CoV, β-CoV, γ-CoV, and Δ-CoV, which cause human diseases. All the identified seven human CoVs (HCoVs), HCoV-NL-63, HCoV-229E, HCoV-OC-43, HCoV-HKU-1, SARS-CoV, MERS-CoV, and SARS-CoV-2 cause respiratory diseases in humans. The emergence of β-CoVs (SARS-CoV in 2002 and MERS-CoV in 2012) demonstrated the emerging possibility of new pathogenic CoVs in humans *via* zoonotic transmission (1, 2). The pathogenic CoV, SARS-CoV-2, causes coronavirus disease 2019 (COVID-19), first reported in Wuhan, China, in late 2019, resulting in severe global public health issues and substantial economic losses (3–5). Bats act as a reservoir for CoVs; therefore, it is speculated that the SARS-CoV-2 originated in horseshoe bats before transmitting to humans *via* an unknown intermediate host (6). Because of its high contagiousness and the presence of asymptomatic human carriers, this pathogen spreads rapidly across the globe, claiming human lives and obstructing social and economic activity (4, 7). According to the WHO 2021 report, over 246.53 million COVID-19 cases and 49.97 million deaths are associated with this disease worldwide (8). The United States has the highest COVID-19 prevalence, with 45.63 million cases and a death rate of approximately 0.74 million, followed by India with 34.29 million cases and 0.46 million deaths, Brazil with 21.80 million cases and 0.61 million deaths, and the United Kingdom with 9.02 million cases and approximately 0.14 million deaths (8).

It is true that the COVID-19 pandemic has dominated both the printed and electronic media and scientific literature. However, other infectious diseases such as tuberculosis (TB), a contagious and airborne bacterial infection caused by *Mycobacterium tuberculosis*, should not be neglected. TB has been an ancient threat to public health since the prehistoric ages and is one of the top 10 causes of human death (9). Further, TB is the second leading infection after COVID-19. According to the WHO 2021 report, nine out of ten people infected with TB belong to the 30 countries with the highest TB burden, including India, China, Indonesia, the Philippines, Pakistan, Nigeria, etc. (10). WHO data from more than 200 countries shows that the number of people who died from TB increased from 1.4 million to 1.5 million (2019–2020). Aside from the increased global death rate, the number of newly diagnosed TB cases reported to the local governments fell from 7.1 million to 5.8 million in the same period, which is an 18% decrease from 2012, with 1.3 million TB deaths (9). This report further stated that among the 9.9 million TB infections last year, 4.1 million were either undiagnosed or not reported to the state in 2020 (9). TB affects males and females of all ages, with adult men bearing the greatest burden, accounting for approximately 56% of all TB cases in 2020, while women and children accounted for 33% and 11%. A higher proportion of TB cases among adult men consistently shows that TB affects adult men more than females, which is most likely due to men’s having a higher detection and reporting rate than females (9).

People affected by chronic respiratory, metabolic, or cardiovascular diseases carry a higher risk of severe COVID-19 infection (11, 12). The COVID-19 signs and symptoms are almost identical to those of TB and other influenza infections. Therefore, SARS-CoV-2 coinfection with other viruses (13), bacteria, and fungi (14, 15) frequently impedes COVID-19 prevention, diagnosis, and control strategies. Both COVID-19 and TB target the human respiratory tract, particularly the lungs, and are transmitted *via* aerosol droplets from an infected person to a healthy one. Evidence shows that COVID-19 patients coinfected with TB (COVID-19-TB) have a higher risk of death than a single pathogen (9, 15). The COVID-19 pandemic has already challenged public health care systems and impaired TB services for TB patients, increasing their morbidity and mortality globally. *M. tuberculosis* interacts with other pathogens such as HIV in coinfection to impair the host’s defenses (16). However, the synergism between SARS-CoV-2 and *M. tuberculosis* is yet unclear. Thus, a comprehensive investigation of COVID-19-TB coinfection’s impact, synergism, and pathogenesis is of great clinical importance (8, 9, 11). There have been few clinical studies on COVID-19-TB coinfection (15, 17, 18), and some of the published case reports and cohort studies have serious flaws. First, the sample sizes are small, and most of the studies were carried out in low-TB-burden countries with poorly described clinical features. The second problem is a lack of understanding of previous comorbidities like diabetes, hypertension, obesity, etc. Sometimes, it is difficult to confirm whether TB was known before or after the COVID-19 diagnosis. Despite these flaws, studies have concluded that active TB makes a patient more vulnerable to severe COVID-19. However, it is worth noting that various social conditions, history of diseases, comorbidities, and limited access to healthcare influence the prognosis of TB patients to COVID-19 coinfection.

The current study aimed to discuss the impact of COVID-19-TB coinfection, the SARS-CoV-2 host cellular receptors (ACE2, auxiliary, and alternative to ACE2 receptors), and pathogenesis. We also discuss *M. tuberculosis* host cellular receptors (TLRs, NLRs, CLRs, scavenger receptors) and pathogenesis. In addition, we highlight the impact of COVID-19 on TB patients and the pathological pathways that link SARS-CoV-2 and *M. tuberculosis* coinfection. Further, we discuss the impact of BCG vaccination on coinfection and the diagnostic problems associated with COVID-19-TB coinfection.

## SARS-CoV-2 and *M. tuberculosis* Host Cellular Receptors and Pathogenesis

### SARS-CoV-2 ACE2, Auxiliary and Alternative to ACE2 SARS-CoV-2 Receptors and Pathogenesis

The SARS-CoV-2 S protein comprises two subunits, S1 and S2, that aid in viral attachment and entry into the host cell cytoplasm. Subunit S1 has a receptor-binding domain that promotes host receptor binding, whereas subunit S2 directly fuses the virus with the host membrane (7, 19). The S1 attaches to the angiotensin-converting enzyme 2 (ACE2) peptidase on the target cell surface and invades *via* clathrin-mediated endocytosis (20–22). Infection of susceptible cell lines (19, 23) and transgenic mice expressing human ACE2 (24) demonstrated that the ACE2 protein in human cells primarily acts as a CoV receptor. Several other researchers have confirmed these findings and reported that ACE2 depleted Vero-E6 inhibited SARS-CoV-2 infection (25), Huh-7 (hepatocyte cell line) (25, 26), Caco-2 (immortal human cell line) (27), and Calu-3 (pulmonary cancerous cell line) (28, 29). On the other hand, SARS-CoV-2 can infect ACE2-deficient cells (30), and the reason behind this infection might be mutations in the spike protein (31). ACE2 exists in two forms, i.e., cell membrane-bound and soluble, which are released after cleavage by the metallopeptidase domain 17 (ADAM17). According to a recent finding, soluble ACE2 forms a complex with the S of SARS-CoV-2 and vasopressin proteins to promote infection (32). SARS-CoV-2 first infects respiratory epithelial and alveolar cells (33), followed by infecting and replicating in ciliated mucus-secreting bronchial epithelial cells (also called type-2 pneumocytes of the lungs) (34), macrophages (35), intestines, heart, kidneys, blood, liver, and brain (7). COVID-19 infection increased *ACE2* expression (threefold) in respiratory epithelial cells (36), owing to the fact that the *ACE2* gene promotes interferon (IFN) production (36). Despite discovering SARS-CoV-2 genomic mRNA in airway epithelial cells that express ACE2 (36, 37), a correlation between SARS-CoV-2 infection and *ACE2* expression at a single-cell level needs further investigation to reveal the viral pathogenesis mechanism. Because SARS-CoV-2 damages multiple organs, it is also possible that other host cellular factors aid in virus replication and transmission *in vivo*. Several SARS-CoV-2 receptors act as cofactors, allowing the virus to enter the host cell cytoplasm. For example, heparan-sulfate polysaccharide expressed on host cell surfaces binds to SARS-CoV-2 spike protein (38, 39), indicating that heparan-sulfate depletion may reduce SARS-CoV-2 attachment and infection (39). Like other influenza viruses, SARS-CoV-2 may use heparan-sulfate to bind to the host surface, increasing viral interactions with other host cellular receptors for entry and pathogenesis (40). This is because many influenza viruses attach to heparan-sulfate due to *in vitro* adaptation to cell culture. Future research should determine whether SARS-CoV-2 binding is a natural viral capability to heparan-sulfate on the host cell. In addition to heparan-sulfate, scavenger receptor B-1 is another host cellular receptor that facilitates the uptake of high-density lipoprotein (HDL). According to the evidence, SARS-CoV-2 S1 shows an affinity for host HDL, and the increased HDL in cells increases viral attachment and infiltration (41). Increased HDL levels may overexpress the *SCARB1* gene (which encodes scavenger receptor B1), resulting in severe SARS-CoV-2 infection. Conversely, *SCARB1* knockdown cells showed reduced infection, indicating that scavenger receptor B-1 facilitates cellular uptake of HDL-bounded SARS-CoV-2. It has been confirmed that SARS-CoV-2 is distinguished from SARS-CoV (42) due to the presence of a unique furin cleavage site at the S1 and S2 domains. Two separate studies (43, 44) found that the polybasic motif at the S1/S2 C terminal binds directly to the neuropilin-1 receptor to promote SARS-CoV-2 pathogenesis. It has been reported that scavenger receptor B-1 and neuropilin-1 receptors may overexpress *ACE2* to promote viral entry into the host cell cytoplasm (41, 43, 44), suggesting that scavenger receptor B-1 and neuropilin-1 act as cofactors that enhance SARS-CoV-2 attachment and penetration *via* ACE2 receptors. In the absence of ACE2, several other potential candidate receptors allow SARS-CoV-2 attachment and penetration into host cells. In a study, receptors such as host cellular tyrosine-protein kinase (30), low-density lipoprotein, and C-type lectin receptors (CLRs) (45) showed strong affinity for the SARS-CoV-2 spike. Depleting these host receptors may reduce infection, whereas overexpression of these proteins in ACE2-knockout cell lines induced SARS-CoV-2 infection, indicating that these cellular receptors and ACE2 have a similar function. Basigin (BSG), also known as CD147 or EMMPRIN, is an alternative widely expressed putative cellular receptor on the human cell surface for SARS-CoV-2 attachment and entry (46), though a study failed to confirm this finding (47). A study reported that human BSG expression in mice allowed for severe COVID-19 infection because SARS-CoV-2 could not infect mice *via* ACE2 (23), implying that BSG may be an alternative to ACE2. SARS-CoV-2 invades and enters the human lungs and avoids detection by the host immune system, infecting and replicating in ciliated mucus-secreting epithelial type-2 pneumocytes (34) and alveolar macrophages (35).

### *M. tuberculosis* TLRs, NLRs, CLRs, and Scavenger Receptors and Pathogenesis

Like SARS-CoV-2, *M. tuberculosis* invades and replicates in ciliated mucus-secreting epithelial type-2 pneumocytes and alveolar macrophages (48, 49) using host pattern recognition receptors (PRRs), complement receptors (CRs), toll-like receptors (TLRs), CD14 receptors, dendritic cell-specific ICAM-3-grabbing-non-integrin-1 (DC-SIGN), Fcγ receptors, mannose receptors, and scavenger receptors (49, 50). Alveolar macrophages phagocytosed *M. tuberculosis* before being transferred to lysosomes for destruction (48, 49, 51). A successful *mycobacterial* infection depends on its encounter with the host cell factors, particularly alveolar macrophages. *Mycobacteria* are gram-positive and have been classified as acid-fast bacilli due to the presence of a lipid-rich cell wall. The physical features of pathogens and host factors significantly affect *mycobacterial* pathogenesis (49, 52). Many immunological peculiarities are attributed to glycolipid layers in the *mycobacterial* wall, including lipoarabinomannan and mycolic acid. *M. tuberculosis* uses various surface molecules (52–54) to bind host surface receptors, including surfactant proteins, Fcγ receptors, CRs, CD14 receptors, and macrophage mannose receptors (51). Several Fcγ receptors bind immunoglobulin-G-opsonized *M. tuberculosis*, allowing the bacteria to enter the host cell, promoting phagosome-lysosome fusion and reactive oxygen intermediates (55, 56). One of the most intriguing aspects of *M. tuberculosis* pathogenesis is the fate of macrophages. *M. tuberculosis* communicates with macrophages *via* its various receptors. *In vivo* responses of macrophages to *M. tuberculosis* receptors are poorly understood, while *in vitro* studies show that specific receptors may influence the macrophage response. For example, Fcγ receptor binding produces reactive oxygen intermediates and phagosome-lysosome fusion, whereas *M. tuberculosis* binding *via* CR3 or mannose receptor inhibits the respiratory burst and prevents phagosome maturation almost identical to the endosome (57), indicating that *mycobacteria* can interact and penetrate the target cell cytoplasm *via* several receptors. After inhaling *M. tuberculosis*, the bacterium activates the alternative pathway and is opsonized by the CR3 within the alveolus, enhancing phagocytosis by alveolar macrophage CR1 or CR3 protein (58, 59). Studies revealed that CR3-deficient mice infected with *M. tuberculosis* showed similar bacteria burden, granuloma formation, and survival rate (60, 61), demonstrating CR3 redundancy. In another study, cholesterol was essential for *mycobacteria* entry into alveolar macrophages because cholesterol-depleted cells inhibited bacterial infection, demonstrating that cholesterol probably mediates the phagosome-associated tryptophan-aspartate proteins, preventing phagosome maturation into phagolysosome (56, 62).

Host surface PRRs, including TLRs, CLRs, NOD-like receptors (NLRs), scavenger receptors (such as MARCO, CD36, and MSR1), aryl hydrocarbon receptors, CD14, and AIM2-like receptors, recognize *M. tuberculosis* pathogen-associated molecular patterns (PAMPs) during phagocytosis (63). Much research has been done on *mycobacteria*-derived PAMPs with TLRs on the host cell surface. In TB patients, TLRs are responsible for recruiting MYD88, TIR adaptor-inducing interferon (TRIF), Toll/IL-1, and TRIF. TLRs have been classified into two groups: endosome localized (TLR3, TLR9, TLR7, TLR8) or surface localized (TLR1, TLR6, TLR2, TLR5, TLR4, TLR10), which help in the recognition of bacterial surface antigens, particularly the LPS layer (64). After pathogen infection, TLR recruits different adapter molecules to relay signals for activating signaling pathways like NF-κB, MAPK, PI3K, and Akt, further inducing pro-inflammatory cytokines and type-1 IFN. TLR expression and activation are useful indicators of the immune response in TB patients (65). Mice lacking the MYD88 signaling adaptor molecule are extremely vulnerable to *M. tuberculosis* infection (66), implying the importance of MYD88 against *M. tuberculosis* infection (66). In another experiment, TLR2 deficient mice showed defective granuloma formation after *M. tuberculosis* infection and were much more susceptible to infection than WT mice. People with TLR2 genetic polymorphisms were more susceptible to pulmonary tuberculosis (67), whereas *TLR4*, *TLR7*, and *TLR8* polymorphisms were more susceptible and severe to TB in Asians, particularly Indians (68), implying that blocking phagosome-lysosome fusion increased phagocytosis in addition to a weak immune response (69). Mice lacking TLR9 expression die soon after *M. tuberculosis* infection (70). Conversely, a study found that only MyD88 (not TLR2/4/9) was a key factor for macrophage activation in TB patients (71). A study also discovered that cytokines could be produced during *M. tuberculosis* infection using TLR or caspase-1 (71). *M. tuberculosis* expresses various lipoproteins to recognize TLRs on the host cell. For example, lipoprotein lpqH recognizes TLR2 (72), while TLR3 promotes an IL10 response *via* the PI3K/AKT signaling pathway in TB patients (73). The leucine-responsive regulatory protein of *M. tuberculosis* regulates the TLR2-mediated PI3K/AKT pathway, inhibiting inflammatory cytokine production and de-regulating macrophage antigen presentation (74). Previously, it was discovered that the *M. tuberculosis*-secreted proteins Mce3E and PtpA target MAPK and NF-κB pathways to modulate TLR signaling (75, 76). *Mycobacterial* phagocytosis into macrophages was also promoted by activating the signaling pathways ERK and MAPK, which are likely to be involved in *mycobacterial* pathogenesis (77). More research is required to investigate the potential molecular interactions of TLR activating signaling pathways with *M. tuberculosis* receptors. Apart from TLRs, several intracellular NLRs, i.e., NOD1, NLRC4, NOD2, and NLRP3, recognize bacterial components and activate inflammatory pathways against invading pathogens (78). In response, *M. tuberculosis* can escape from phagosomes *via* an ESAT-6 system-1 (ESX-1)-associated pathway (79). In contrast, a study found that NOD2 deficient mice increased susceptibility to *M. tuberculosis* infection (80). Simultaneously, activation of NOD2 in alveolar macrophages with muramyl-dipeptide derived from the *mycobacterial* cell wall prevents bacterial movement of autophagy-related polypeptides to the autophagosome, implying the importance of PRR in autophagy (81). In another study, three *NOD2* gene polymorphisms in African American people were linked to TB susceptibility (82). It has also been reported that the *NOD2* single-nucleotide polymorphism showed increased susceptibility to TB (83). Many recent studies have looked into the roles of other NLRs, particularly the NLRP3 inflammasome, during *M. tuberculosis* infection. *M. tuberculosis* ESAT-6 system activates the NLRP3 inflammasome in macrophages, resulting in the production of IL1-β and pyroptosis (84). The *NLRP3* gene, an adaptor protein, and caspase-1 mediate immune responses against *M. tuberculosis* infection in mice. In the presence of NLRP3, caspase-1, and PYCARD, *M. tuberculosis*-infected macrophages showed induced IL1-β, indicating that IL1-β production makes mice more susceptible to *M. tuberculosis in vitro*. However, *in vivo*, NLRP3, Casp-1 depleted, and WT mice produced the same amount of IL1-β during *M. tuberculosis* infection (85). Thus, *M. tuberculosis* may activate the inflammasomes to promote persistent TB. Despite discovering the preventive role of novel NLRs against *M. tuberculosis*, more research is needed to explore the NLR-induced signaling pathways. Further, it is unknown whether *M. tuberculosis* effectors interact with NLR domains (e.g., the PYD domain) to regulate signaling pathways. In the future, it will be fascinating to investigate the potential underlying regulatory mechanisms in host cells that control NLR signaling pathways in TB patients. Based on phylogeny and structure, CLRs have been classified into 17 groups, including collectins, endocytic, selectin receptors, proteoglycans, and phagocytic receptors. The CLRs identified include mannose receptor, mincle, DC-SIGN, dectin-1-3/macrophage, dendritic cell immune receptor (DCIR), and collectin CL-L1, CL-K1 are all critical factors for *mycobacterial* invasion. Important immune regulators are CLRs that bind to *M. tuberculosis* carbohydrates, lipids, or proteins. In addition, CLRs directly recognize *M. tuberculosis* mannose-capped lipoarabinomannan (ManLAM) and cord factors and are essential immune modulators in TB patients (86), apart from promoting *mycobacterial* phagocytosis by alveolar macrophages (87). *Mycobacteria* that target DC-SIGN generate intracellular signals to stimulate dendritic cells to produce IL10, implying that the *mycobacteria*-targeted DC-SIGN suppresses the host immune response during infection (88). Trehalose 6, 6′-dimycolate, and cord factor are lipid-rich cell wall components in virulent *mycobacteria*, which act as a macrophage-inducible CLR in humans (89). Evidence shows that Dectin1 and TLR2 can regulate pro-inflammatory macrophages against invading *mycobacteria* (90). In another study, Dectin1 contributes to *M. tuberculosis* susceptibility in mice (91). In contrast, Dectin2 acts as a receptor for *M. tuberculosis* ManLAM to prevent infection (92). Surprisingly, both Dectin-3 and Mincle are required for the host immune response, with Dectin-3 stimulating Mincle expression and thus amplifying the mincle-mediated immune response against *M. tuberculosis* (93, 94). Collectin CL-LK was a novel soluble CLR capable of binding to *M. tuberculosis* ManLAM (95). In addition, Dectin-2 and DCIR modulate immune responses against *M. tuberculosis* by sustaining IFN-1 signaling in dendritic cells (96). More research on human CLRs recognizing *M. tuberculosis* components and their interactions with other immune cells is needed to gain insight into *M. tuberculosis*-induced innate immunity.

## Impact of SARS-CoV-2 Pandemic on TB Patients

The Global TB Network reported COVID-19-TB coinfection in several countries (15, 97, 98). Both pulmonary and extrapulmonary TB (disseminated TB: evidence of TB in bone, central nervous system, gastrointestinal and genitourinary tracts, larynx, lymph nodes, peritoneal, pleural, spinal cord, etc.) patients have been found coinfected with COVID-19 (15, 99). Eight studies reported 80 COVID-19-TB coinfected humans from nine countries, with Italy reporting the highest cases (51%) of active pulmonary TB (100). Some of the COVID-19-TB coinfections reported in different countries are shown in **Table 1**. Belgium, Spain, Brazil, Singapore, France, Switzerland, Italy, India, Russia, and China reported COVID-19-TB coinfections. Due to the recent COVID-19 rise, we can expect increased COVID-19-TB infections in people of all races, ages, and genders. According to clinical evidence, COVID-19 occurs with or without TB, i.e., before, after, or during concurrent TB (126). COVID-19-TB coinfection was more common in migrants and men, i.e., > 80% of male cases (98, 127). Both COVID-19 and TB are highly infectious diseases (128); for example, a single COVID-19 patient may infect approximately 2.5 people in only five days, whereas an active pulmonary TB patient may infect upto 15 people per year (may be due to long incubation period) (129). The reported predisposing factors in COVID-19-TB patients were comparable to those in TB patients without COVID-19. Diabetes, kidney failure, liver disease, and smoking are comorbidities for COVID-19-TB infections (15). A study found that 41% of the COVID-19-TB patients were smokers, 31% were unemployed, and 20% had a history of alcohol use (98, 127). According to human studies, COVID-19 in TB patients is more common, particularly in high-TB burden countries, such as India, Vietnam, etc. (130), while Brazil and Argentina, with high COVID-19 cases, experienced varying degrees of healthcare system disruption (131). Similarly, COVID-19-TB cases within a country differ depending on people’s socioeconomic status and disease preventive measures. In addition, several COVID-19-TB risk factors, such as age, malnutrition, and comorbidities such as pre-respiratory disorders, diabetes, etc., have been identified (126). Studies also revealed that older and younger people, with or without pre-existing clinical complications, a previous history of TB or lung injury, are at risk of COVID-19 infection (132). A clinical study revealed a similar dysregulated immunological response in COVID-19-TB patients, implying that coinfection poses a dual risk of disease worsening (132). In addition, poor hygiene, overcrowding, and other autoimmune diseases are risk factors for developing both diseases (133, 134). A study that developed a model of pathogen dissemination showed that high-risk influenza patients are at high risk of *M. tuberculosis* infection (135). The WHO reported that the COVID-19 social and economic losses could be more severe in the highest TB burden regions (136). Another serious issue of COVID-19 is TB nature, patient’s long treatment (usually 6–24 months), poor treatment outcomes (i.e., drug-resistant TB), drug discontinuation, and other stringent pandemic isolation measures (137). Treatment discontinuation risks and other issues confronting TB clinical trials in COVID-19 cases have raised concerns (138). To address these issues, self-administered anti-TB therapy monitoring with digital technology or video-assisted administration has been recommended (139). Hypoxemia, respiratory disorders, glucose abnormalities, prolonged hospitalization, superimposed bacterial infection, and multiple organ failures have been reported in COVID-19-TB cases (98, 140).

**Table 1:** Studies assessing the impact of the COVID-19 pandemic on TB patients.

Country	Number of COVID-19-TB cases	Clinical features	Main outcomes	Ref
**A multinational study, including Belgium, Brazil, France, Italy, Russia, Singapore, Spain, Switzerland**	49 COVID-19-TB patients	COVID-19 cases: 5 asymptomatic, 43 symptomatic, (36 pulmonary TB, 13 extrapulmonary TB)	After treatment, 18 recovered, 25 were under treatment with follow-up, while six patients died	(15)
**China**	Three COVID-19-TB cases	Respiratory distress, hypoxia, hemoptysis, low peripheral blood count, reduced immunity, CT scan showed significant ground-glass opacities in lungs	After 14 days of treatment, all three patient’s conditions were stabilized and discharged	(101)
**China**	Three COVID-19-TB cases	Fever, cough, chest tightness/pain, dyspnea	After 24–37 days of treatment, all three patients were recovered and discharged	(97)
**China**	15 COVID-19-TB cases	Fever, cough, dyspnoea, chest pain, headache, fatigue	After treatment, ten patients recovered while the rest five died	(102)
**China**	Four COVID-19-TB cases	Fever, cough, chest distress, myalgia, shortness of breath	One died while the rest three health status was recovered	(103)
**China**	Eight COVID-19-TB cases	Fever, fatigue, sputum production, cough, dyspnoea	After treatment, all eight patients were recovered	(104)
**China**	Nine COVID-19-TB cases	Fever, fatigue, cough, dyspnea, chest tightness	Four severe and five mild cases were hospitalized for treatment	(105)
**China**	Three COVID-19-TB cases	Fever, cough, fatigue, wheeze, weight loss, etc.	One severely ill patient died of respiratory and circulatory failure, and two recovered	(106)
**India**	Single COVID-19-TB case	Fever, cough, shortness of breath, etc.	Anti-TB medications; however, the patient’s health status deteriorated with increased dyspnoea worse respiration and died	(107)
**India**	Single COVID-19-TB case	Fever, cough, chills, night sweats, loss of appetite, chest pain, breathlessness	The patient was given anti-TB treatment and is under treatment	(108)
**India**	Single COVID-19-TB case	Headache, dizziness, vomiting, CNS TB	The patient was discharged after 28 days of anti-TB medications; he was asymptomatic on the second visit after two weeks. Rifampicin, isoniazid, and pyridoxine were continued	(109)
**India**	22 COVID-19-TB cases	Fever, cough, breathlessness	After treatment, sixteen cases recovered and were discharged follow-up, while six died	(110)
**America**	Single COVID-19-TB case	Fever, cough, hypertension, diabetes, atrial fibrillation, increased leukocytosis, high inflammatory markers, CRP, IL6, LDH, ferritin, fibrinogen	After 51 days of anti-TB drugs treatment, the patient was recovered and discharged	(111)
**America**	Single COVID-19-TB case	Fever, headache	Death of brain herniation	(112)
**Italy**	69 COVID-19-TB cases (60 were migrants and 9 were Italian)	The majority were elderly with comorbidities, hypertension, prostatic hypertrophy, liver disease, fever, cough, vomit, etc.	After treatment, eight died, and 61 recovered and were discharged with follow-up	(98)
**Italy**	20 COVID-19-TB patients	Fever, cough, chest pain, and dyspnoea were common among patients	After treatment, twelve recovered along with a case with chest pain, and vomit was unchanged, while seven severe cases were treating	(113)
**Brazil**	Two COVID-19-TB cases	Fever, cough, mild respiratory distress, myalgia, headache	After anti-TB medications for one week without giving antiretroviral therapy, all the patients were clinically stable and discharged with follow-up	(114)
**Bangladesh, India, Nepal**	Six COVID-19-TB cases	Fever, fatigue, cough, myalgia	After treatment, patients were recovered and discharged with follow-up	(115)
**Philippines**	113 COVID-19-TB patients	Of the total, 22 suffered from hypertension, 14 diabetes, 5 cancers, 8 cardiac diseases, 4 asthma, and 3 COPD	Of the 70 hospitalized, 22 non hospitalized, and 21 unknown, 32 died, and 57 recovered	(116)
**South Africa**	115 COVID-19 with HIV 510 COVID-10 without HIV	Most individuals suffer from hypertension, diabetes, chronic kidney disease, chronic lung diseases, previous TB, current TB, HIV, etc.	of the 115 COVID-19 diseased combined with HIV, 42 had previous TB history and 16 current TB, whereas, among 510 COVID-10 without HIV, 45 had previous TB history and 10 current TB	(117)
**Haiti**	Single COVID-19-MDR-TB case	Isoniazid and rifampin resistance was evident; chest radiograph revealed a large lobe cavity with lobe opacity; before hospitalization and treatment, the patient left and was not followed up	unknown	(118)
**Singapore**	Four COVID-19-TB cases	Fever, cough, dyspnea, pleuritic chest pain	After treatment, the patient was stabilized and discharged	(119)
**Panama**	Two COVID-19-TB cases	Pneumonia, confusion, urea, respiratory rate, blood pressure, mild neutrophilia, anemia, and CRP, ferritin, and procalcitonin levels were increased	After 14 days of treatment, both patients recovered and were discharged with follow-up	(120)
**France**	Single COVID-19-TB case	Fever, acute/severe respiratory disorders	After 14 days of treatment, the patient recovered and discharged	(121)
**South Africa**	Single COVID-19-TB case	Fever, cough	The patient received oral prednisone doses and is under-observation	(122)
**South Africa**	Single COVID-19-TB case	Hydrocephalus, arterial ischemic stroke, extensive cerebral sinus venous thrombosis, induced pro-inflammatory cytokines response, d-dimers, fibrinogen, and ferritin	Required neuro-rehabilitation for a month. after treatment, the patient was recovered, discharged with home occupational and physiotherapy	(123)
**Saudi Arabia**	Single COVID-19-TB case	Hypertension, diabetic, severe/acute pneumonia	After anti-TB medications for four months along with oxygen supportive care, the patient recovered and was discharged with followed-up	(124)
**Turkey**	Single COVID-19-TB case	Fever, cough, elevated neutrophil count, creatinine, d-dimer levels	After administration of multi-task clinical management approaches (including antiretroviral and anti-TB medications), the patient recovered	(125)

COVID-19 and TB are the two leading causes of death among respiratory diseases (12, 141). In a cohort of 49 COVID-19-TB cases from eight countries, i.e., Brazil, Singapore, Russia, Spain, Switzerland, Belgium, France, and Italy (15), 26 patients were detected as TB positive before COVID-19, and COVID-19 was diagnosed in 14 patients before TB treatment. Of the total, 42 patients had active pulmonary TB, and seven developed TB complications. Similar findings were confirmed by a study conducted in India (110). In a similar study conducted in Sondalo Hospital, Italy, clinical, laboratory, and radiological characteristics showed that SARS-CoV-2 infected 20 of the 24 hospitalized TB patients. Four patients received only hydroxychloroquine at the time of hospitalization. A single TB case later coinfected with COVID-19 died, whereas the remaining 19 people experienced severe medical outcomes such as pneumonia (113). In a case-control study from Shenyang Primary-Care Hospital, China, 13 out of 36 COVID-19 people tested positive for TB (142), implying that *M. tuberculosis* infection increases host susceptibility to SARS-CoV-2 infections. Therefore, routine TB coinfection diagnosis in COVID-19 cases is advised. Moreover, a large-scale clinical research study should evaluate the negative impact of severe TB on COVID-19 coinfection. The mortality rate was higher in aged people (> 70 years old) and COVID-19-TB patients, whereas the migrants had lower mortality rates, which may be attributed to their younger age and the absence of clinical comorbidities (15, 98). COVID-19 is associated with higher mortality among TB patients (98, 140) who acquired COVID-19 through nosocomial transmission. According to a meta-analysis, COVID-19 coinfection increases the risk of TB patient death (1.4 times) (143). The findings of 69 patients from eight countries (98) suggest a COVID-19-TB fatality rate of 11.6% and 14.3% (15). Mortality is likely to occur in old COVID-19-TB patients having medical comorbidities. For example, 5% of the old cases with comorbidities in Italy (113). According to evidence, migrants had lower mortality rates and comorbidities, most likely due to their younger age (144). However, patients with severe TB or MDR-TB could experience increased mortality, particularly younger individuals (144). A longitudinal cohort study looked at the risk of COVID-19 patient deaths when they were also infected with TB, and it was discovered that the COVID-19-TB death rate was higher (2.17 times) than the single COVID-19 death rate. In comparison, nearly 25% of COVID-19-TB patients recovered, which was lower than single COVID-19-infected individuals (116), highlighting the need to prioritize routine TB testing for COVID-19 patients. To understand this coinfection interaction, the Global TB Network and WHO jointly launched a study on TB and COVID-19 patients. COVID-19-TB clinical comorbidities have been described in 597 cases studied from 132 centers in 36 different countries/states (145).

Studies have also elucidated the molecular interactions of the host with COVID-19-TB, and it was found that this viral-bacterial coinfection worsens respiratory disorders (12, 141). Clinical manifestations such as hemoptysis, cough, weakness, and fever are common among people suffering from COVID-19-TB, making an accurate diagnosis difficult. As COVID-19-TB causes an unbalanced inflammatory response in severely infected individuals; therefore, understanding the molecular interactions between SARS-CoV-2-*M. tuberculosis* and their host will be critical for developing anti-COVID-19-TB therapeutic agents (141). A study also examined the immunological status in COVID-19-TB cases, i.e., increased C-reactive protein (CRP), d-dimers, ferritin, neutrophils, lymphocytes, cytokine storm, and chemokines have been linked to COVID-19 severity and patient mortality (146, 147). The bronchoalveolar fluid from severe/mild COVID-19 patients showed increased chemokine (C-C motif ligand-2: CCL-2) and CCL-7, attracting the CCL2-associated monocytes. In addition, severe COVID-19 patients revealed increased mononuclear phagocyte counts (accounting for 80% of total bronchoalveolar fluid cells) compared to 60% in mild cases and 40% in healthy controls (147, 148). Phagocyte activation can induce pro-inflammatory cytokines (cytokine storm), which increases alveolar epithelial infection (149). Increased pro-inflammatory cytokine, IL6, IL1-β, and IP10 expression in COVID-19 patients promotes neutrophil proliferation and infiltration into the lung for injury (150, 151). Severe TB patients showed increased pulmonary immune cell (macrophages, dendritic cells, etc.) responses. These immune cells overproduce cytokines like IL1, IL10, IL18, IFNα, and IL6 (152, 153). In brief, COVID-19 coinfection with TB promotes cytokine storms that cause multiple organ injuries, particularly to the lungs, heart, or liver (5, 150, 151). These two pathogens induce cytokine storms with similar features but different magnitudes and outcomes. For example, *M. tuberculosis* infection elicits a lower immune response than SARS-CoV-2. In addition, cytokine storm causes respiratory distress in COVID-19 patients, whereas it causes long-term organ damage/failure in chronic cases. In addition to cytokine storm, lymphocytopenia (decreased lymphocyte counts) is another notable feature of the immune response in COVID-19 and TB patients. Lymphocyte counts of less than 1.5×10^9^ per liter have been observed in severe COVID-19 cases (154, 155). After penetration, SARS-CoV-2 may directly invade lymphocytes and destroy them. The damaged lymphatic system in severe COVID-19 patients further promotes lymphocytopenia (156). Increased pro-inflammatory cytokine levels of IL6 and TNFα can also cause lymphocytopenia in severe COVID-19 patients (156). Moreover, T lymphocyte dysregulation was observed in TB cases. For example, a study also reported that the host defense response is dependent on CD4+ cell responses against *M. tuberculosis* infection (157, 158). A low CD4+ cell count increases the risk of TB reactivation, resulting in severe radiological lesions or patient death (48). Immunosuppression caused by CD4+ cell depletion in SARS-CoV-2-TB cases may increase the disease severity and patient morbidity. Therefore, prolonged clinical consideration should be given to all the above concerns.

## Pathological Pathways That Connect SARS-CoV-2 and *M. tuberculosis* Coinfection

**Figure 1:**
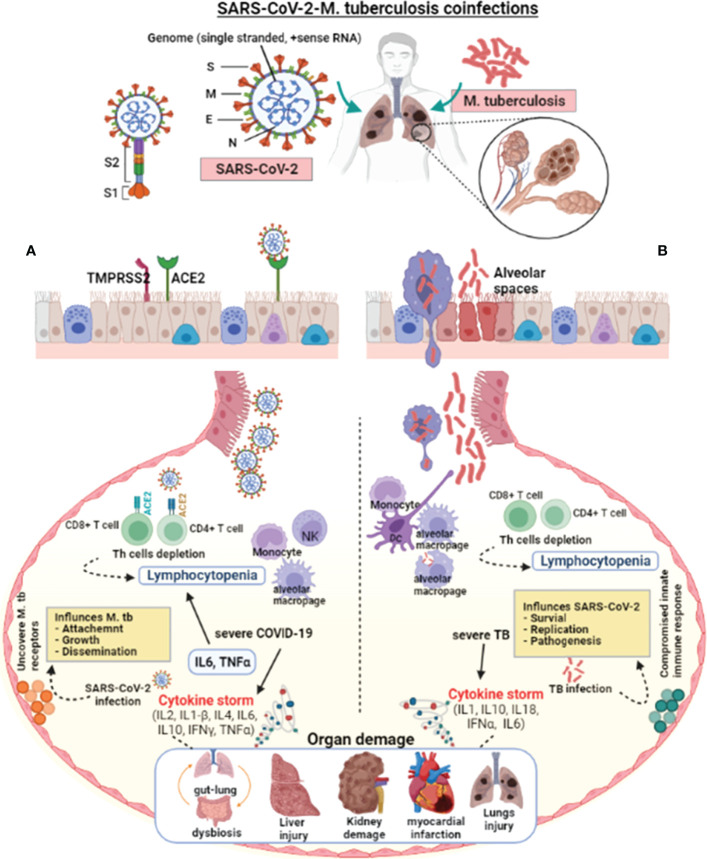
Pathophysiological effect of SARS-CoV-2 and *M. tuberculosis* on the host cell. SARS-CoV-2 enters the host *via* aerosol, travels to the alveoli, and interacts with the host’s innate immune cells. SARS-CoV-2 and *M. tuberculosis*-infected alveolar macrophages secrete cytokines to activate other immune cells, i.e., monocytes, macrophages, CD4+, CD8+ lymphocytes, neutrophils, dendritic cells, and natural killer cells to the infected site. **(A)** In severe COVID-19 infections, the exuberant pro-inflammatory cytokine response may result in lung injury. The lungs of severely infected COVID-19 patients showed an elevated immune response, which resulted in pneumonia, respiratory distress, lung fibrosis, and lymphocytopenia (decreased lymphocyte count). SARS-CoV-2 virulence factors interact with the host lungs, eliciting an immune response. These interactions may weaken the innate immune response, leading to increased *mycobacterial* attachment, growth, and dissemination. **(B)** In severe TB infection, activated lymphocytes produce excessive pro-inflammatory cytokines response called cytokine storms. Infection with *M. tuberculosis* causes symptomatic TB in people who have weakened immune systems or are immune-compromised. Cytokine storm-mediated inflammation causes multiple organ dysfunctions. *M. tuberculosis* infection and colonization may predispose the lungs to SARS-CoV-2 by down-regulating the host immune responses, allowing virus survival, growth, and pathogenesis. The suppressed host immune response in COVID-19-TB coinfection may cause exacerbated TB. In addition, reactivation of latent to active TB indicates that SARS-CoV-2 infection can exacerbate *M. tuberculosis* pathogenesis.

Since the first SARS-CoV-2 outbreak in Wuhan city (133), there has been little data on *M. tuberculosis* coinfection, probably due to *M. tuberculosis’s* prolonged incubation from exposure to symptoms (134, 159). Based on the population study findings and the published data about the etiology of COVID-19 and TB, it is possible to discuss some aspects of COVID-19-TB coinfection (160). Both COVID-19 and TB have airborne transmission primarily affecting the lungs and share the same social determinants and symptoms (**Figure 1**). However, SARS-CoV-2 and *M. tuberculosis* present significant differences in their pathogenesis, and learning about their interactions with the host may aid in the development of new COVID-19-TB treatment strategies. SARS-CoV-2 and *M. tuberculosis* may act synergistically in infected host cells (141). During latent TB infection, *M. tuberculosis* interacts with the pulmonary microenvironment and induces immune responses (141). Concrete evidence suggests that SARS-CoV-2 may cause hostile pro-inflammatory responses, including IL2, IL1-β, IL4, IL6, IL10, IFNγ, and TNFα in the infected cells (133). In addition, several stimuli probably add up in COVID-19-TB coinfection, leading to a cytokine storm. The necrosis and pyroptosis of the lung may cause damage-associated molecular pattern dispersion. SARS-CoV-2 presents a much more aggressive pyroptosis and promotes immunopathology and tissue damage (161, 162). Pulmonary alveoli are like battlefields for both SARS-CoV-2 and *M. tuberculosis*. *M. tuberculosis* silently infiltrates the lungs and avoids an exaggerated host immune response. In the case of mild infection, individual immune responses successfully eliminate both pathogens (163). Sometimes, the damaged lungs of TB patients influence local immunity and make the host more susceptible to COVID-19 and other airborne pathogens (15). It has been shown that SARS-CoV-2 aggravates the pulmonary TB status, causing latent TB to become active, which further deteriorates lung function (164). Further, inflammatory cytokine responses play a vital role in host resistance to *M. tuberculosis* infection as revealed in murine models (165), which was also validated in TB patients with mutations (blocking) in the IFNγ and IL12 signaling pathways (165, 166). According to the findings of a cohort of 49 COVID-19 TB patients, the possibility that SARS-CoV-2 infection may increase the occurrence of TB coinfection (15). A meta-analysis showed that patients with a TB history are not more likely to contract COVID-19; however, TB may increase the risk of contracting COVID-19 (143). Active TB patients in an Italian hospital had higher COVID-19 coinfection and clinical characteristics (113). COVID-19 coinfection worsened TB status and increased death in a group of 69 patients (98). In contrast, 20 COVID-19-TB coinfected people had benign clinical manifestations, with only one death. A chest X-ray revealed that TB lesions had not been aggravated; only four of the patients had recently developed pneumonia. In a quantitative study conducted in Belarus, 844 COVID-19 confirmed patients admitted to hospitals were tested for TB. Of the total, 47 patients had TB and were resistant to rifampicin (167). A study including 36 COVID-19 cases from China discovered that a history of pulmonary TB increases a patient’s vulnerability to severe SARS-CoV-2 infection (142), implying that TB status should be routinely checked in COVID-19 cases (135). According to findings from a developed pathogen model, populations at high risk of contracting SARS-CoV-2 may have a higher *M. tuberculosis* prevalence (135).

## Impact of BCG on COVID-19 Patients

Previous immunological studies have shown that BCG vaccination prevents TB, DNA, and RNA viral infections, such as herpes and influenza viruses, resulting in lower morbidity and mortality (168, 169). The BCG vaccine stimulates the host immune response to produce antibodies that protect against TB infection and prevent the spread of the invaded *M. tuberculosis* (170). BCG vaccination in adults may increase non-specific pro-inflammatory cytokines IL1-β and IL6 against other bacterial pathogens (171) by stimulating CD4+ and CD8+ cells against non-targeted antigens, modulating lymphocyte responses against secondary infections. BCG administration also promotes innate immune cell responses, including monocytes, natural killer cells, and alveolar macrophages, increasing host resistance to future bacterial/viral infections (172, 173). For example, BCG vaccination induces a protective humoral immune response against the negative-strand RNA pneumovirus that causes respiratory disorders (174). Based on previous research, the BCG vaccination may boost the host’s immunity to reduce the severity of COVID-19 (175). Subsequently, increased pro-inflammatory cytokines TNFα, IL1-β, IL6, IFNγ, alveolar macrophages, T-lymphocytes, and antibody titers have been observed in BCG vaccinated people (168). Based on the host immune responses, BCG vaccination may prevent or reduce SARS-CoV-2 infection, particularly in BCG vaccinated children (176, 177). Although these findings are hypothetical, a nationwide BCG program may reduce COVID-19 severity and mortality; however, clinical trials on large datasets are recommended for BCG’s efficacy against the COVID-19 pandemic. Following BCG administration, healthy people will experience an immune response to SARS-CoV-2 infection, likely reducing viral loads, inhibiting viral replication, reducing pro-inflammatory cytokine responses, and inducing lymphocytopenia (176). Australia’s Muldron Children’s Research Institute started a phase III trial to determine if a healthcare worker’s BCG immunization program affects SARS-CoV-2 infection. National BCG vaccination programs in countries like Asia, Africa, and America have resulted in fewer COVID-19 cases (177, 178), necessitating further investigation to reveal the BCG connection with mild COVID-19 infections. BCG vaccination most probably stimulates innate immune responses against SARS-CoV-2. Following BCG vaccination, epigenetic programming trains monocytes and natural killer cells to clear many viral infections, particularly SARS-CoV-2 (176). More clinical data is required to understand the trained innate immune response and the adverse effects of potential innate and adaptive immunity in BCG-vaccinated COVID-19 cases. However, increased immunosuppression and cytokine storms were linked to severe complications in COVID-19 patients (179). Given the increased global TB and SARS-CoV-2 burden, particularly in developing and underdeveloped countries, BCG vaccination that could aid in the fight against both TB and COVID-19 would be highly desirable. Previously, recombinant adenoviral vectors were designed to boost patient immunity with humoral and cellular immune response enhancement (180). Adults with or without prior BCG vaccination should receive ChAdOx1-85A (181). In addition to BCG vaccination, scientists are struggling to develop an effective SARS-CoV-2 therapeutic agent to treat severe cases, particularly people who have pre-comorbidities. Isoniazid, ethambutol, pyrazinamide, and rifampin drugs are used to treat resistant TB (98, 140), whereas bedaquiline, clofazimine, levofloxacin, and linezolid can be combined with pyrazinamide for the treatment of MDR-TB cases. COVID19-TB coinfection can be treated with azithromycin, hydroxychloroquine (an anti-rheumatic drug), and protease inhibitors (lopinavir, ritonavir, darunavir, etc.) (15, 98). Moreover, enoxaparin and parnaparine anticoagulants may prevent the formation of blood clots (15).

## Diagnostic Challenges and Management of COVID-19-TB Cases

Many governments imposed lockdowns to prevent SARS-CoV-2 transmission (182). People were forced to stay indoors, which affected their lives and health. Because of the similarity in symptoms between COVID-19 and TB, most people probably experience delayed TB with almost similar symptoms to COVID-19. In addition, the COVID-19-TB coinfection may have discouraged people from getting a diagnosis even if they experienced disease symptoms. These undiagnosed people were mostly from the region with lower socioeconomic status, struggling to make ends meet and eat. As a result, their pre-existing misery would have been exacerbated by the additional fear of quarantine. Furthermore, COVID-19-TB transmission could be a major concern due to close interactions at home.

The implementation of city lockdowns delayed TB diagnosis among those seeking medical advice, as non-emergency services were suspended in most parts of the world. Along with the restriction on accessing government and private clinics, laboratories were primarily dedicated to processing COVID-19 patient specimens (183), resulting in decreased COVID-19-TB detection and notification, as demonstrated by the 2020 Global TB Report. According to this report, the patient reporting rate was reduced by 25% in the three highest TB burden countries (Indonesia, India, and the Philippines) in only six months (January to June 2020) compared to 2019. COVID-19 has disrupted TB services globally, particularly in countries with the highest TB prevalence. For example, data from sixteen countries showed that COVID-19 disrupted TB services, particularly during the first four months of the pandemic (184). According to reports, deaths from TB are expected to increase by almost 13% in the coming years (185), which is probably a significant setback in the global fight against global TB. Due to COVID-19 restrictions, patients who have already been diagnosed with TB will also suffer. Sputum microscopy and bacterial culture growth primarily used for pulmonary TB follow-up patients were lost due to lockdowns. As a result, those with anti-TB therapy failure or multi-drug resistance to TB may have continuous health deterioration (183). Counseling and patient motivation are required to cope with TB clinical consequences and long-term anti-TB medication. Both COVID-19 and TB primarily affect the human lungs; however, TB symptoms shortly appear after COVID-19 infection (186, 187). Given the clinical complications of COVID-19 and TB, including cough, fever, breathing problems, and lung lesions (186, 187), proper diagnostic facilities are encouraged to avoid the misdiagnosis of one disease over the other. Tuberculin tests are less expensive and more widely used, whereas IGRAs are rarely used to diagnose TB (188). However, due to host immunity against *M. tuberculosis* or the BCG vaccine (189), a potential error in disease diagnosis may exist in immune-compromised people (190). Increased age, lymphocytopenia, and immunosuppressive therapies have also been linked to false-negative IGRAs (181), resulting in a false TB diagnosis. Furthermore, excessive inflammatory marker production may interfere with the IGRA testing, and increased CRP levels may confound the false-negative QuantiFERON-TB Gold In-Tube Test (191). In another study, severe COVID-19 patients had a lower peripheral lymphocyte count, natural killer cells, high CRP levels, and pro-inflammatory cytokines IL6, IL8, TNFα, IL2-R, IL1-β, etc. This unusual cytokine production may destroy host immune responses, i.e., low lymphocyte infiltration into infected lungs and multiple organ damage (192). Because SARS-CoV-2 is a novel CoV and has no specific treatment (5), suspected people must be tested accurately to prevent virus transmission. Apart from traditional diagnostic testing for detecting viral antigens or antiviral antibodies, the nucleic acid detection method has been developed for routine testing (193). To identify COVID-19 clinical manifestations (such as fever, dyspnea, cough), radiological features, and rapid virus transmission among people (194), doctors may encounter difficulty in differential diagnosis or overlook TB. In addition, scientists are struggling to develop an effective SARS-CoV-2 therapeutic agent to treat severe cases, particularly people who have pre-comorbidities. A lack of TB-specific radiological findings is likely to be missed due to the non-specific COVID-19-TB features. Another reason could be immunomodulators (a class of drugs that target pathways to reduce immune response) in mild/severe COVID-19 cases that may reactivate latent TB in endemic areas (107). Based on clinical, radiological, and laboratory findings, a patient in Russia developed pulmonary TB in the infiltration phase and COVID-19, which was complicated by spontaneous pneumothorax of the left lung. A TB specialist, an infectious diseases specialist, and a thoracic surgeon discussed the case condition. Due to his severe condition, he was given detoxification, bronchiolitis, and antibiotics (azithromycin, ceftriaxone, levofloxacin). Following TB confirmation, the patient was started on anti-TB antibiotics; rifampicin, ethambutol, and pyrazinamide. The chest drain was removed from the left pleural cavity, and lung function quickly recovered. The postoperative wound area had residual subcutaneous emphysema. Two nucleic acid tests for SARS-CoV-2 yielded negative results, and the patient was transferred to a TB hospital for further management (195).

Access to COVID-19 and TB diagnostic testing is a critical first step toward reducing disease transmission. As a result, there is a case to improve access to COVID-19 and TB testing through the implementation of simultaneous testing, particularly in countries with a high TB burden, to reduce the impact of the current pandemic on TB patients. Several countries, including Indonesia (196), South Africa (197), Nigeria (198), and India (199), began concurrent diagnostic testing for COVID-19 and TB during the pandemic. Notably, India’s Ministry of Health and Family Welfare issued guidelines to reduce the impact of the COVID-19 pandemic on TB control and management, including COVID-19 screening for TB confirmed patients or TB screening for COVID-19 confirmed patients (199). FIND, a non-profit global alliance, develops low-cost diagnostic tests for COVID-19 and TB. The project allows for rapid integrated testing for COVID-19 and TB for improved detection of both diseases. Rapid antigen testing for COVID-19 or TB symptoms assists in patient management by identifying coinfection hotspots. In addition, the private healthcare sector collaborated with the Joint Effort for the Elimination of TB (JEET) to improve access to affordable TB and COVID-19 testing services and educate people about coinfection precautions and symptoms and treatment. These experiences have demonstrated the importance of concurrent COVID-19 and TB testing in increasing access to coinfection diagnostics. The difficulties encountered during the simultaneous COVID-19-TB testing were primarily related to staff deficiencies and a lack of personal protective equipment. Huge gaps exist in the area of simultaneous COVID-19-TB testing, for example, the development of integrated COVID-19-TB testing for the same sample would increase the integrated system’s cost-efficiency. Other key areas that require additional research include evaluating a community for simultaneous testing and the cost-effectiveness of simultaneous testing (200). Future efforts should include the development and validation of COVID-19 and TB-related symptom-screening apps, considering factors like geography, age, risk factors, and research into the optimal sampling strategy for COVID-19-TB testing.

## Conclusions and Future Perspectives

In the current scenario, TB coinfection should always be suspected in COVID-19 patients, whether or not they exhibit specific respiratory symptoms”– the data presented above indicate that TB coinfection contributes to COVID-19 severity and worse outcomes. Thus, rather than all patients, COVID-19 and TB coinfection may be suspected in severe TB patients, those resistant to therapy, or those from countries with a high prevalence of TB infection.

The COVID-19 pandemic has significantly affected human life and substantially lost the global economy. Available data shows a high mortality rate among COVID-19-TB coinfected individuals. SARS-CoV-2-*M. tuberculosis* coinfections impair host immunity. It is reasonable to assume that COVID-19 and TB harmful synergism contribute to severe clinical manifestations, most likely affecting patients, causing respiratory disorders *via* cytokine-mediated responses and an increased risk of latent TB reactivation.

To avoid COVID-19 coinfection, severe TB patients should only be hospitalized. Despite increasing COVID-19-TB cases, comprehensive global clinical trials are required to investigate the potential effect of COVID-19 on TB cases deeply. The current COVID-19 cases undermine the WHO goal of reducing the global TB burden. To lessen the impact of the COVID-19 pandemic on TB patients, consistent practices are needed to highlight the global TB burden while taking urgent steps to support and organize innovative TB control programs and minimize hospital visits to prevent COVID-19 coinfection (201). TB patients’ follow-up in their homes should be encouraged to ensure proper anti-TB medications. In addition, the implementation of smartphone technology might be an excellent choice for assessing and monitoring anti-TB medications. Collaboration of WHO anti-TB programs with local governments in developing countries is also recommended to prevent COVID-19-TB coinfection. Increasing testing capacity helped the healthcare system in high-risk areas. Implementing effective strategies to identify new TB hotspots and ensure continuous anti-TB drug transportation and distribution will be beneficial, especially in high-risk areas (202). Advanced preventive measures can also be taken to control COVID-19-TB coinfection. Despite the COVID-19 pandemic’s social and economic challenges, proper healthcare systems, effective social policies, and equal health facilities should be provided to control and prevent TB coinfection.

## Author Contributions

XX, ZB, and TS performed the original draft preparation and revision. ZS, and NY made suggestions for the writing of the manuscript and revision. All authors actively contributed to the article and approved the submitted version.

## Funding

This study was supported by grants from the major science and technology special project of Yunnan Province, No. 2019ZF004.

## Conflict of Interest

The authors declare that the research was conducted in the absence of any commercial or financial relationships that could be construed as a potential conflict of interest.

## Publisher’s Note

All claims expressed in this article are solely those of the authors and do not necessarily represent those of their affiliated organizations, or those of the publisher, the editors and the reviewers. Any product that may be evaluated in this article, or claim that may be made by its manufacturer, is not guaranteed or endorsed by the publisher.
